# Lumos: Software for Multi-level Multi-reader Comparison of Cardiovascular Magnetic Resonance Late Gadolinium Enhancement Scar Quantification

**DOI:** 10.1007/s10278-025-01437-2

**Published:** 2025-03-17

**Authors:** Philine Reisdorf, Jonathan Gavrysh, Clemens Ammann, Maximilian Fenski, Christoph Kolbitsch, Steffen Lange, Anja Hennemuth, Jeanette Schulz-Menger, Thomas Hadler

**Affiliations:** 1 Working Group on Cardiovascular Magnetic Resonance, Experimental and Clinical Research Center (ECRC), a joint cooperation between the Charité – Universitätsmedizin Berlin and the Max-Delbrück-Center for Molecular Medicine, Berlin, Germany; 2 Charité – Universitätsmedizin Berlin, corporate member of Freie Universität Berlin and Humboldt-Universität zu Berlin, ECRC Experimental and Clinical Research Center, Lindenberger Weg 80, 13125 Berlin, Germany; 3 DZHK (German Centre for Cardiovascular Research), partner site Berlin, Berlin, Germany; 4 Department of Cardiology and Nephrology, HELIOS Hospital Berlin-Buch, Berlin, Germany; 5 Physikalisch-Technische Bundesanstalt (PTB), Braunschweig and Berlin, Germany; 6 Department of Computer Sciences, Hochschule Darmstadt - University of Applied Sciences, Darmstadt, Germany; 7 Charité – Universitätsmedizin Berlin, corporate member of Freie Universität Berlin and Humboldt-Universität zu Berlin, Berlin, Germany; 8 Fraunhofer MEVIS, Bremen, Germany; 9 Deutsches Herzzentrum der Charité (DHZC), Institute of Computer-assisted Cardiovascular Medicine, Augustenburger Platz 1, Berlin, Germany

**Keywords:** Cardiovascular magnetic resonance, Late gadolinium enhancement, Comparison software, Scar quantification, Quality assurance

## Abstract

Cardiovascular magnetic resonance imaging (CMR) offers state-of-the-art myocardial tissue differentiation. The CMR technique late gadolinium enhancement (LGE) currently provides the noninvasive gold standard for the detection of myocardial fibrosis. Typically, thresholding methods are used for fibrotic scar tissue quantification. A major challenge for standardized CMR assessment is large variations in the estimated scar for different methods. The aim was to improve quality assurance for LGE scar quantification, a multi-reader comparison tool “Lumos” was developed to support quality control for scar quantification methods. The thresholding methods and an exact rasterization approach were implemented, as well as a graphical user interface (GUI) with statistical and case-specific tabs. Twenty LGE cases were considered with half of them including artifacts and clinical results for eight scar quantification methods computed. Lumos was successfully implemented as a multi-level multi-reader comparison software, and differences between methods can be seen in the statistical results. Histograms visualize confounding effects of different methods. Connecting the statistical level with the case level allows for backtracking statistical differences to sources of differences in the threshold calculation. Being able to visualize the underlying groundwork for the different methods in the myocardial histogram gives the opportunity to identify causes for different thresholds. Lumos showed the differences in the clinical results between cases with artifacts and cases without artifacts. A video demonstration of Lumos is offered as supplementary material 1. Lumos allows for a multi-reader comparison for LGE scar quantification that offers insights into the origin of reader differences.

**Supplementary Information:**

The online version contains supplementary material available at 10.1007/s10278-025-01437-2.

## Introduction

Cardiovascular magnetic resonance imaging (CMR) is an increasingly important tool in clinical routine and research with high prognostic value [1]. It is currently the gold standard for the assessment of cardiac structures and volumes, and especially used for noninvasive myocardial tissue characterization [2]. CMR offers late gadolinium enhancement (LGE) sequences, which are currently the gold standard for detection of myocardial fibrosis [2–4].

**Fig. 1:**
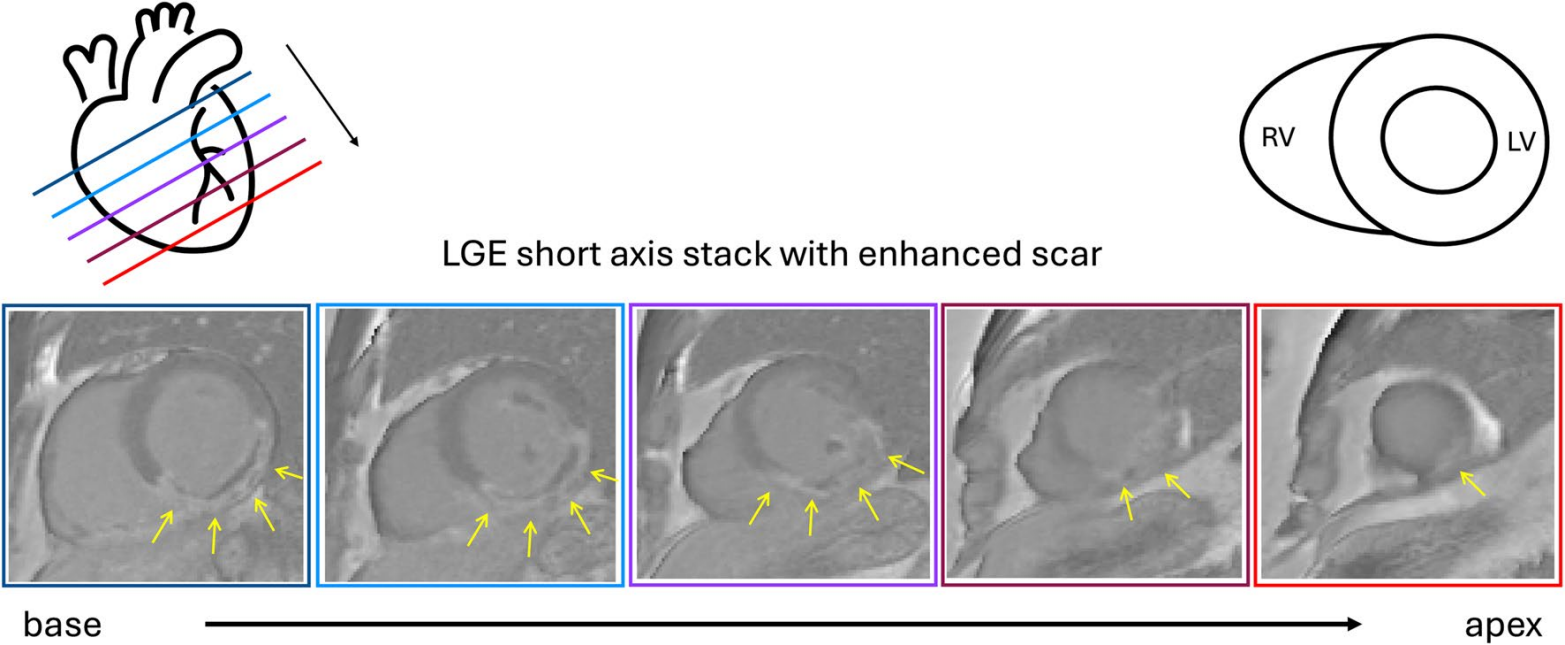
Shown here is the short-axis view of an LGE stack from base to apex. The yellow arrows indicate where fibrosis is present. The colored boxes indicate the position of the slice in reference to the heart on the top left. The top right shows the structure of the heart. Legend: RV: right ventricle, LV: left ventricle

LGE is a CMR technique where the patient is given a gadolinium-based contrast agent before the image acquisition [5–7]. The contrast agent remains in the damaged tissue of the myocardium longer than in the healthy [5]; thus, fibrotic scar tissue appears brighter compared to the healthy tissue. Figure 1 shows how the image stack is acquired as cross sections of the heart from base to apex [2]. The enhanced scar within the myocardium is marked by the yellow arrows. Whereas LGE is the gold standard for the detection of fibrosis, quantification of fibrosis is still not included in clinical post-processing workflows since the uncertainty between readers and methods is high [1].

**Fig. 2:**
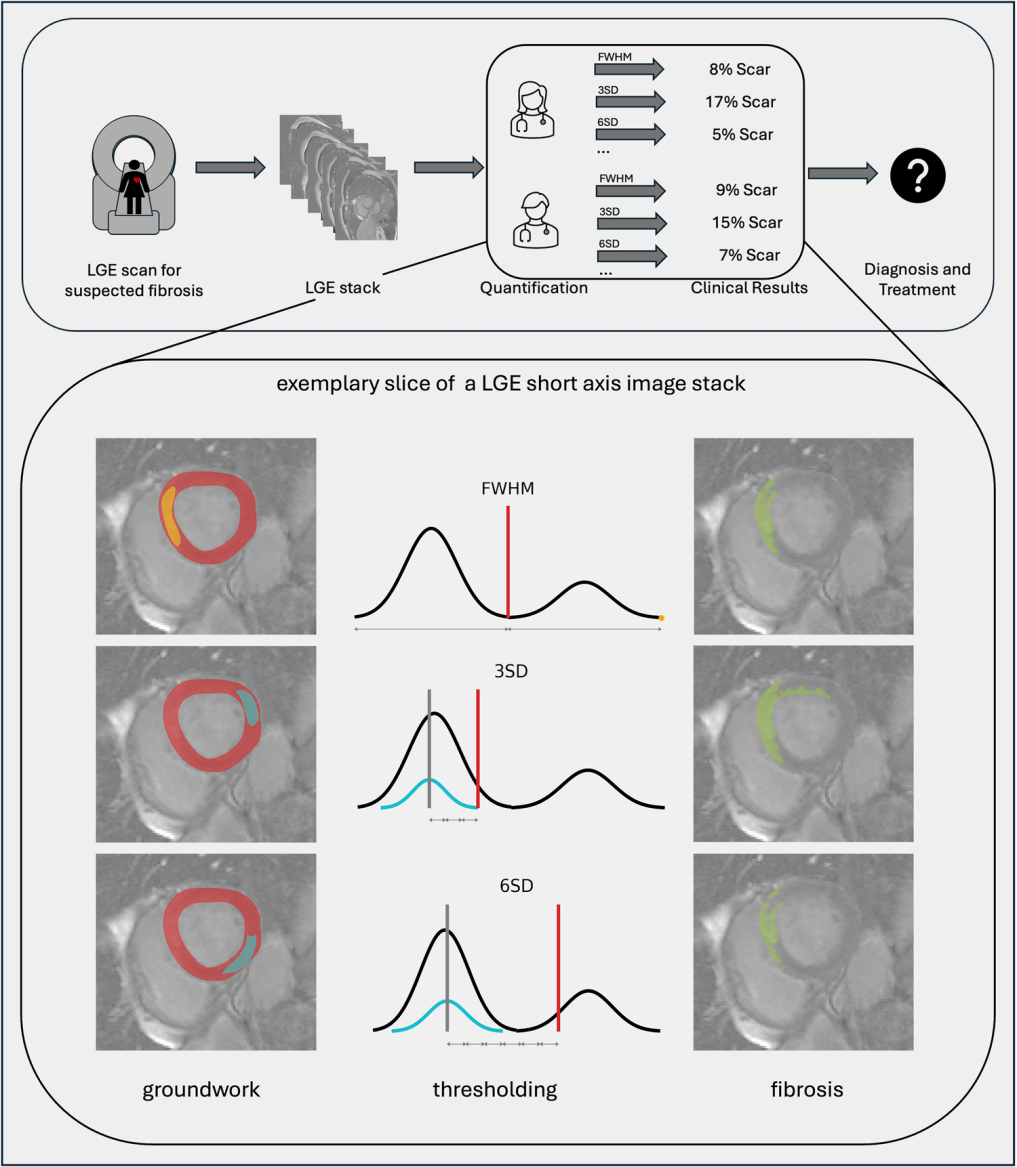
Uncertainty of scar quantification: After the patient is scanned and an LGE image stack is acquired by obtaining short axis images that show the cross sections of the heart from the base to the apex, different readers would quantify the scar using different methods. The groundwork for the thresholding methods is the annotation of the left myocardium (red) in the short-axis images and a region of interest (ROI) (orange) for FWHM or a remote myocardial region (light blue) for the *n*SD method. The thresholding then determines the scar mass. Different methods and different groundwork lead to varying clinical results

This uncertainty can be seen in Fig. 2: A patient with suspected fibrosis is examined by an LGE CMR scan. When quantifying scar, it is done slice-by-slice on a short-axis (SAX) image stack by using a quantification method. Most quantification methods use the pixel intensities in the myocardium of the left ventricle as a basis [8, 9]. In a healthy person, the histogram of the pixel intensities of the myocardium is one distribution, depending on the scanner and coils used; it could be either Rician or a non-central $\upchi$-distribution [10]. In the case of fibrosis, the given contrast agent remaining in the damaged tissue enhances the pixel intensities in these parts of the myocardium [5]. That means instead of one distribution, one expects to acquire two overlapping distributions: one representing the healthy tissue on the left-hand side and one representing the enhanced, damaged tissue on the right-hand side. Thresholding methods are applied with the goal to find the threshold that optimally separates these two distributions. A simplified view of the distributions can be seen in the middle column of the lower part of Fig. 2.

While there are different methods available to quantify the fibrotic tissue, mainly used in research are thresholding methods like full width half maximum (FWHM) or *n*-standard deviations from remote (*n*SD) [11, 12]. As illustrated in the lower part of Fig. 2, the FWHM method calculates the threshold between those two distributions by using a region of interest (ROI) in the infarcted myocardium. It takes the brightest pixel which is part of the ROI and the darkest pixel of the myocardium and sets the threshold in the middle of the detected pixel intensities [13]. The *n*SD method utilizes a remote myocardial region in the healthy myocardium as a referential basis of the threshold calculation. It works under the assumption that the remote myocardial region is normally distributed and calculates the mean and standard deviation for that remote region [8]. The threshold is then estimated as the mean plus a fixed number *n* of standard deviations [8]. Typically, *n* is between two and six [12, 14, 15], but *n* between one and nine have been tested [8, 9, 16].

There is little agreement on which of the methods works best depending on the entity of the fibrosis [8, 9, 12, 17]; additionally, they are all influenced by different confounders, such as the reader (person applying the method), how the myocardium is annotated (which supplies the groundwork on which the thresholding methods are used), as well as the placement of the ROI and remote myocardium, size of the remote myocardium, scanner properties, and image quality, etc. [1, 7, 8]. The thresholds can differ a lot depending on these confounders [8] (Fig. 2). These threshold differences directly influence the percentage of fibrotic tissue, and the different scar sizes would lead to unclear or even wrong diagnoses in clinical practice.

The aim of this project is to develop, implement, and test a multi-level multi-reader comparison tool that addresses the need for quality assurance in LGE scar quantification. This tool should be able to analyze different confounders for LGE scar quantification methods as to how they impact the variability and reproducibility of scar tissue assessments. To this end, the software development and components of Lumos are elaborated, the geometrical approaches to modeling scar quantification, and finally, Lumos’ utility is demonstrated with two experiments.

## Materials and Methods

First, the software foundation Lazy Luna [18, 19] will be explained, and necessary add-ons will be explained. Next, information on how the data needed to be preprocessed and on the implemented rasterization algorithm will be given. Then, the setup and connection between the different tabs in the graphical user interface (GUI) will be explained, and lastly, the data and analysis which were utilized as evidence for Lumos usability will be provided.

### Software Foundation Lazy Luna and How It Was Extended

Lumos was developed as an open-source extension of the open-source two-reader comparison software Lazy Luna [18, 19]. Lazy Luna is a published open-source software developed for comparison of postprocessing results of CMR cine and mapping images on the level of annotations or clinical parameters between two readers.

Lumos was built on Lazy Luna’s backend already developed by Hadler et al. [18, 19], in which image data and annotations are processed to calculate clinical parameters and efficiently use the processed information for comparison on multiple levels: statistical, case-wise, and metrical.

Image data is required in the Digital Imaging and Communications in Medicine (Dicom) format [20, 21] and annotations as json files. The annotations include myocardial contours, the annotation of the ROI or remote myocardial region, the detected scar, and exclusions for each image in the LGE image stack available for the case. The annotations are saved as polygons or multipolygons depending on the annotation type.

For data storage and backend utility and efficiency, a mongo DB database was used where information was stored in the following structured classes for a more efficient use of the backend: dicoms, annotations, cases, cohorts, image_organizers, evaluations, and task_environments.

“Dicoms” store the local path where the image is stored and some information from the dicom tags. “Annotations” store unique IDs that identify the respective Dicom images and the (multi)polygonal information necessary to plot annotations onto the respective images. “Cohorts” store which cases were uploaded within the study cohort. “Image_organizers” store information regarding the images in one stack, i.e., organizing the slices in a stack from basal to apical by SOP Instance UID. The “evaluations” utilize the information from the "image_organizers" to calculate clinical parameters implemented for the respective image type and connect the clinical parameters with the respective task. A task was defined by the reader (person or artificial intelligence annotating), the method, and the time interval enabling inter- and intra-reader comparisons as well as inter- and intra-method comparisons. The “task_environments” store the information regarding the tasks such as the software used for annotating and the study UIDs of the cases that were annotated, thus connecting the annotations with the images for each task.

As an extension of Lazy Luna to LGE images, clinical parameters such as myocardial mass in grams, scar mass in grams and percentage, exclusions volume in milliliters, scar mass before tissue was excluded in grams, number of slices per image stack, number of slices where a ROI or remote myocardial annotation was added, and the sum of the areas of the remote myocardium in square millimeters were implemented as can be seen in Table 1.

**Table 1:** Clinical results for LGE provided per case by Lumos’ backend

Clinical results [unit]
Myocardial mass [grams]
Scar mass [grams]
Scar percentage [percent]
Exclusions volume [milliliters]
Scar mass before exclusions [grams]
Number of slices per image stack [#]
Number of slices where a ROI or remote myocardial annotation was added [#]
Sum of the areas of the remote myocardium [square millimeters]

The backend of Lazy Luna [18, 19] was implemented in Python 3.12.1. The packages pydicom [22], shapely [23], and rasterio [24] were used for dicoms, annotations, and masks, respectively. For the comparison of two tasks, different metrics were implemented. The Dice score (DSC) and Hausdorff metric (HD) were included. The GUI for Lazy Luna [18, 19] was based on the python package PyQt6 [25, 26]. Figures were included using matplotlib [27] and tables using panda’s DataFrame [28].

As an addition to the backend of Lazy Luna [18, 19] and, as an LGE-specific metric, the threshold difference (TD) was implemented with $TD={t}_{1}-{t}_{2}$, where ${t}_{1}$ and ${t}_{2}$ are the respective thresholds for the compared tasks 1 and 2. Secondly, the area difference per threshold step (ATDT) was implemented to measure the influence of the threshold when calculating the scar:

$$\text{ATDT}=\frac{{\text{area}}_{{\text{scar}}_{1}}-{\text{area}}_{{\text{scar}}_{2}}}{{t}_{1}-{t}_{2}}$$

Where ${\text{area}}_{{\text{scar}}_{1}}$ and ${\text{area}}_{{\text{scar}}_{2}}$ are the areas of the scars detected by tasks 1 and 2, and ${t}_{1}$ and ${t}_{2}$ are the respective thresholds.

### Preprocessing and Rasterization

To utilize the backend described above, it was necessary to implement a preprocessing step to generate exact scar annotations as multi polygons. For that, the different thresholding methods and an exact rasterization algorithm supplying pixel masks from the (multi) polygonial shapes were implemented. The masks were needed to connect the annotation with the image pixels and thus calculate thresholds and scars. They were also used to generate weighted histograms.

The FWHM method was implemented as follows: the threshold is calculated by using the brightest pixel intensity inside the ROI (enhanced myocardium) and then going halfway back towards the darkest pixel intensity inside the myocardium:

$${t}_{\text{FWHM}} = {b}_{\text{ROI}} -\frac{1}{2}\left({b}_{\text{ROI}}-{d}_{\text{myo}}\right)$$

where ${t}_{\text{FWHM}}$ is the threshold, ${b}_{\text{ROI}}$ is the brightest pixel intensity inside the ROI, and ${d}_{\text{myo}}$ is the darkest pixel intensity inside the myocardium. The threshold of the *n*SD method was implemented as follows:

$${t}_{n\text{SD}} = {m}_{\text{remote}}+n{\text{SD}}_{\text{remote}}$$

Here, ${t}_{n\text{SD}}$ is the threshold, ${m}_{\text{remote}}$ is the weighted mean of the remote myocardium, $n\in N$ is the number of standard deviations used, and ${\text{SD}}_{\text{remote}}$ is the weighted standard deviation of the remote myocardium. The weighted mean [29] is calculated as

$${m}_{\text{remote}}=\frac{{\sum }_{i}{x}_{i}{w}_{i}}{{\sum }_{i}{w}_{i}}$$

where ${x}_{i}$ is the pixel intensity for a pixel in the remote myocardium, and ${w}_{i}$ is the weight of that pixel. Correction for the bias in the calculation of the standard deviation was achieved by using reliability weights [29]:

$${\text{SD}}_{\text{remote}}=\sqrt{{\sum }_{i}{\left({x}_{i}-{m}_{remote}\right)}^{2}\left(\frac{{\left({\sum }_{i}{w}_{i}\right)}^{2}}{{\left({\sum }_{i}{w}_{i} \right)}^{2}-\left({\sum }_{i}{w}_{i}^{2}\right)}\right)}$$

Where ${x}_{i}$ is the pixel intensity for a pixel in the remote myocardium, ${w}_{i}$ is the weight of that pixel, and ${m}_{\text{remote}}$ is the weighted mean of the remote myocardium.

The scar multi polygon was then generated by taking all pixels, which are part of the myocardial polygon and have a pixel intensity bigger than or equal to the threshold (Fig. 2, lower part).

**Fig. 3:**
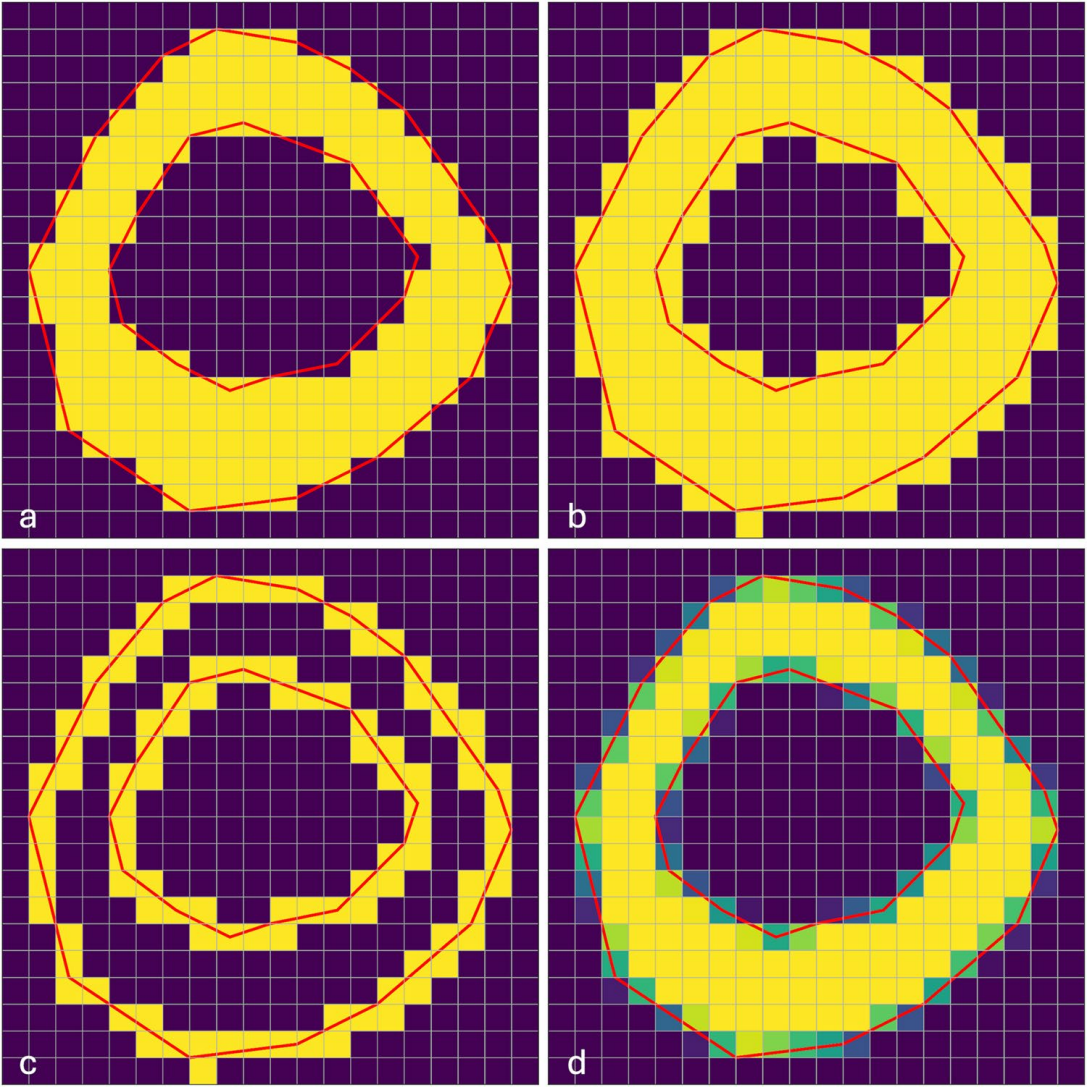
Different rasterization approaches for a polygon which has a shape similar to the myocardial annotation for short-axis images. The polygon is depicted by the red lines, which bound the shape interiorly and exteriorly. The yellow pixels show what is included wholly in the mask generated by the different rasterization approaches: **a** non-exact rasterization with inclusion of all pixels with centroid inside the polygon, **b** rasterization with inclusion of all pixels touched by the polygon shape, **c** pixels on the boundary of the polygon (all pixels touched by the red boundary lines), and **d** exact rasterization with percental inclusion. Yellow pixels are wholly included. The darker the pixel, the less of it is inside the polygon

An exact rasterization algorithm (compare [30]) was implemented using rasterio.features.rasterize [24] as a basis. The weights are also used in the distribution of the histograms later. Figure 3 shows different rasterization algorithms on the example of a polygon in the shape of a mathematical annulus (i.e., the characteristic shape of the myocardium in the short axis view). The rasterization algorithm in Fig. 3a includes pixels in the rasterization mask if the centroid of the pixel is inside the polygon. Note that the area of the yellow mask is close to the exact area of the polygon bounded by the red lines. This is due to the symmetric properties of the myocardial shape. But by not including all pixels that are at least partly inside the polygon, not all pixel intensities inside are found. In Fig. 3b, all pixels that are touched by the same polygon are included, which leads to an inclusion of all pixel intensities inside the polygon, but an overestimated area. As a first step of the exact rasterization algorithm, all pixels in the boundary of the mask from Fig. 3b are determined, as seen in Fig. 3c. Now for each of these pixels, the percentage which is inside the polygon is calculated. This way, an exactly weighted rasterization mask is achieved, as depicted in Fig. 3d. This yields all pixel intensities inside the polygon and the exact weights for the area and histogram calculation. Using the exact weights in the histograms was important to not introduce an additional confounder for the threshold calculation, which for most methods is based on the histograms.

For each method, the preprocessing utilizes the groundwork annotations of the myocardium and the ROI/remote myocardial region. It generates the corresponding masks and automatically calculates thresholds and scar multi polygons per slice and stores them in a dictionary together with the groundwork polygons for the myocardium and the ROI or remote myocardial reference. The exclusions are also stored as a multi polygon. Only the pixel parts in the exclusions multi polygon which intersect with the scar are included. As a result, (multi) polygons based on exactly rasterized masks are generated in json files per method and image which can be imported in Lumos and be viewed in the GUI.

### GUI of the Multi Comparison Tool Lumos

The GUI of Lumos is organized in the following way: first opened is a selection tab where the user can choose between one and twenty previously set methods. An additional sorting tab was necessary to find cases belonging to each of the chosen tasks in the database. In a first overview tab, the user can choose the view (e.g., LGE short axis images) and exclude and include cases. Then, the user can either open a statistical tab (which includes all previously included cases into the statistical calculations) or a case tab for one of the included cases.

**Fig. 4:**
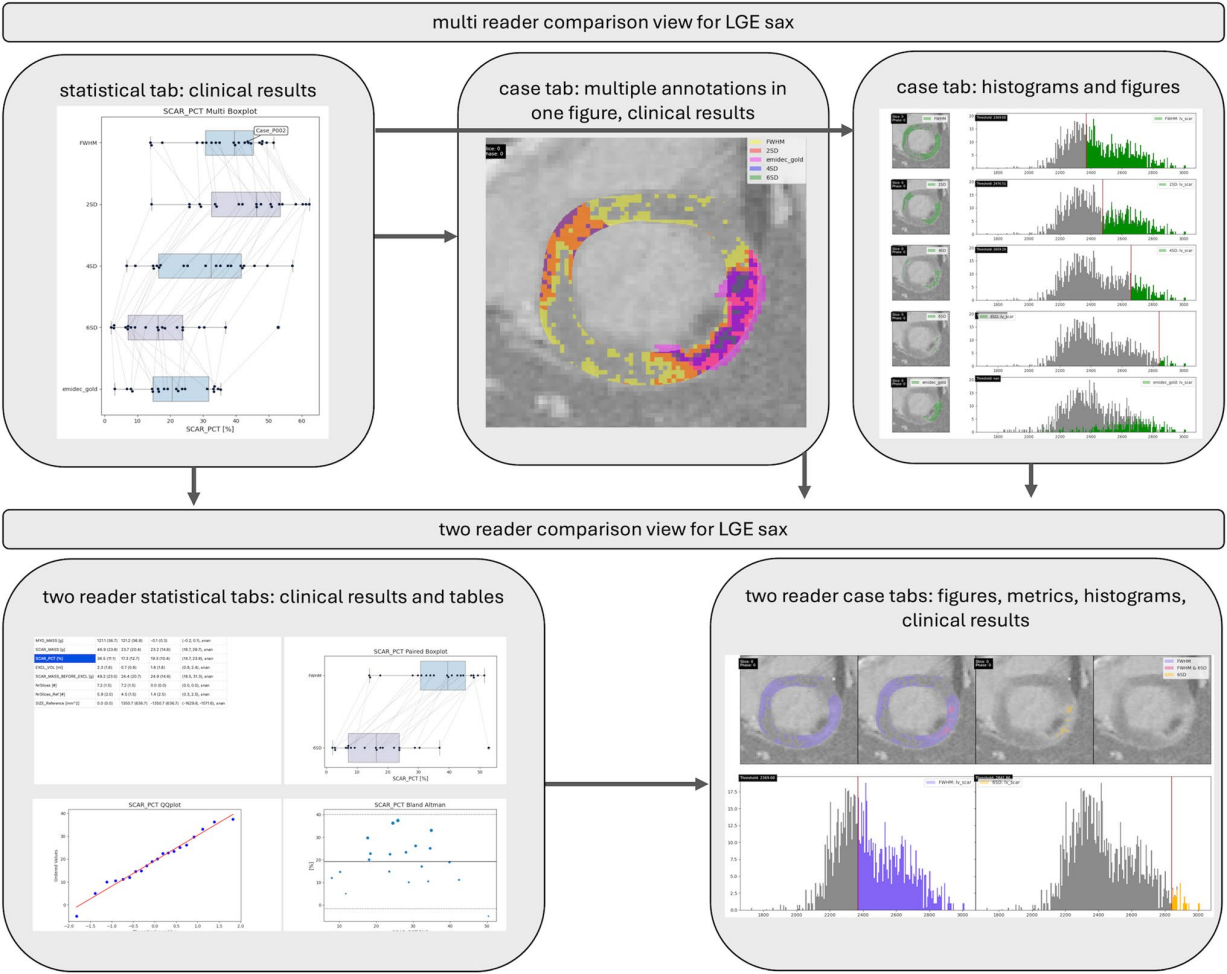
Outlines the different tabs for the multi comparison tool Lumos and the connections between them. On the upper level, the multi comparison tabs (statistical and individual case tabs) are shown and on the lower level, the two-reader comparison tabs. The arrows show the connections between the different tabs

The statistical tab offers multi boxplots for the implemented clinical results for all chosen tasks. Additionally, each case is represented by a point, and lines between the respective case points were implemented to track changes through different methods. The case tabs offer histograms and figures of the dicom images with the respective annotations plotted on top to compare the different annotations. The histograms show the myocardial pixel intensities in gray with regions like the scar or the ROI or remote myocardium highlighted by color accordingly. The user can choose which regions become visible. From the multi comparison tabs, it is possible to go back to the two-reader comparison tabs with the additional possibility of a metrical comparison of two tasks. An overview of the connections between the tabs is depicted in Fig. 4.

The code is available in an open repository on GitHub (https://github.com/thequadsquad/Lumos).

### Data and Analysis

To prove Lumos usability and ability to track different confounders, the open-source EMIDEC dataset [31, 32] was used in two experiments — an *application experiment*, where 20 cases were annotated with different methods and statistically and individually compared, and an *illustration case*, where variations of the same methods for one case were added to show how Lumos can track additional confounders especially when utilizing the same myocardial annotation as groundwork. The statistics presented for the application experiment are not intended to validate a clinical study, but to demonstrate Lumos ability to visualize statistical differences and track these differences back to their respective confounders.

The EMIDEC dataset contains LGE cases in the nifty format, which were converted into dicom format. The offered ground truth annotations of the myocardium and the scar as the agreement of two CMR experts’ (a cardiologist with 10 years of experience and a biophysicist with 20 years of experience, as described in EMIDEC [31]) manual annotations were converted into json files and used as the gold standard.

For the *application experiment*, 20 cases (10 including artifacts and 10 without) had additional groundwork annotations for the FWHM and *n*SD method added by a CMR beginner using the software circle cvi42 (Version: Release 5.13.7) [33]. A CMR beginner is defined here as a reader with no previous experience in postprocessing CMR with CVI42, but who received training on 10 LGE cases before annotating the EMIDEC cases. The detection of artifacts was done visually by the same CMR beginner. A case was considered to include artifacts if an artifact was visible in at least one slice. The idea was to demonstrate Lumos’ usability by comparing annotations of a CMR beginner with some training to the experts’ gold annotations. In order to demonstrate Lumos’s ability to detect confounding factors, a CMR beginner intentionally produced varying annotations.

In the following paragraph, more details on the *application experiment* are given: For the *n*SD method, the myocardium and remote myocardial regions were annotated as additional groundwork. Exclusions were added for 3SD and 6SD. The scar and exact exclusions for different *n* were calculated utilizing the 3SD exclusions for $n=2, 3, 4$ and the 6SD exclusions for $n=5, 6, 7$ and the preprocessing described above. The same myocardial and remote myocardial regions were used as a basis for all *n*SD scar calculations. For the FWHM method, the ROI was annotated starting out on the same myocardial annotations as the *n*SD methods; slight adjustments on the myocardial annotation were made afterwards in three cases.

The preprocessing software was used as described before to generate exactly rasterized (multi) polygons for the different annotation types. The reader EMIDEC gold was defined as one task; the reader CMR beginner combined with each utilized method respectively was defined as the others.

The following clinical results were calculated for the 20 cases of the EMIDEC dataset and the tasks EMIDEC gold, FWHM, and 2-7SD utilizing Lumos backend: the myocardial mass in grams, the scar mass in grams, the percentage of the scar in relation to the myocardium in percent, the exclusion volume in milliliters, the scar mass before exclusions made in grams, the number of slices belonging to the case stack, the number of slices in which an ROI or remote myocardial annotation was placed, and the size of the remote myocardial annotation as a sum for the whole case in square millimeters. The numbers can be seen in Table 4 for the scar percentage as an exemplary clinical result for the methods gold, FWHM, and 2SD-7SD. An overview table of all tasks and clinical results is given in the appendix as supplementary material 2.

Lumos’ aim was to visualize and track the influence of underlying annotation variations on the scar size and other case-related parameters. As an *illustration*, for one case, five versions (V1-V5) of the methods FWHM, 2SD, 3SD, 4SD, 5SD, and 6SD were implemented based on the same myocardial annotation but differently located ROIs and differently located and sized remote myocardial regions. The number of ROIs/remote myocardial regions per case was also varied. To this end, the CMR beginner annotated the myocardium a second time and included different annotations of the ROIs or remote myocardial references. Exclusions were added for the FWHM versions and the 4SD versions. The respective 4SD exclusions were then used for all nSD methods per version. The definitions of the versions can be seen in Table 2. Additionally, a semi-automatic FWHM approach was done on the EMIDEC gold myocardial annotations, utilizing the EMIDEC gold scar annotations as the ROI. For more details on the underlying annotation of versions V1–V5, see supplementary material 3.

**Table 2:** Different versions for the methods FWHM and 2SD-6SD for case P043

	**FWHM**	*n***SD**
**V1**	First attempt, ROI in each slice, no checking if brightest pixel is included or not	First attempt, medium sized remotes (ca. 1/3–1/2 of myocardium), no remote in apical slice
**V2**	Second attempt, different ROI positions in darker regions than V1, ROI in each slice	Same as V1, but added a remote in the apical slice, remotes in each slice
**V3**	ROI in each slice; by manually finding the brightest pixel in the myocardium for each slice, the ROI was put in a position that includes the brightest pixel	Small remotes (ca. 1/6–1/4 of myocardium) as parts of the remotes in V2, remotes in each slice
**V4**	Same as V3, but deleted the ROI in the apical slice	Small remotes (ca. 1/6–1/4 of myocardium) in different positions than V3, remotes in each slice
**V5**	Only one ROI in the midventricular slice was annotated, which includes the brightest pixel	Big remotes (at least 1/2 of myocardium), remotes in each slice

## Results

The multi-level multi-reader comparison software Lumos was successfully implemented and calculated clinical results for the used data. On one hand, the multiple versions of methods for the extra *illustration case* and on the other the results for the *application experiment* containing 20 cases with one version of each method.

### Illustration of Confounding Annotation Variations Effect

Table 3 shows selected clinical results for case P043 for the methods FWHM V2 (brightest pixel not in ROI), FWHM V4 (brightest pixel in ROI), 2SD V3 (small remote myocardial reference), 2SD V5 (big remote myocardial reference), 5SD V3 (small remote myocardial reference), 5SD V5 (big remote myocardial reference), and FWHM as a semiautomatic approach on the EMIDEC gold myocardial annotation utilizing the EMIDEC gold scar annotation as an ROI and EMIDEC gold. Highlighted by the red box are the percentages of scar tissue per method. Note that the results between the same methods vary heavily when different ROIs/remote references were used, e.g., 2SD increased from 23.6% (V5) to 41.7% (V3) when utilizing a small reference instead of a big one. Similar effects can be seen for the FWHM method and 5SD.

**Table 3:**
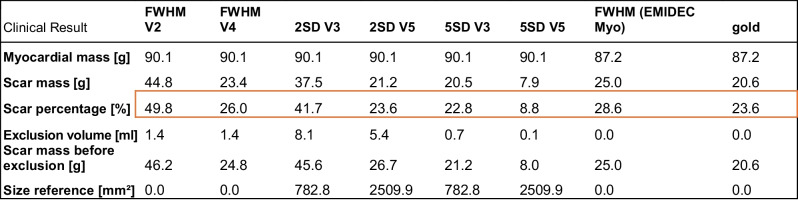
Selected clinical results provided by Lumos’ backend for the case P043 and selected methods

#### Influence of Size of Remote Myocardial Region

**Fig. 5:**
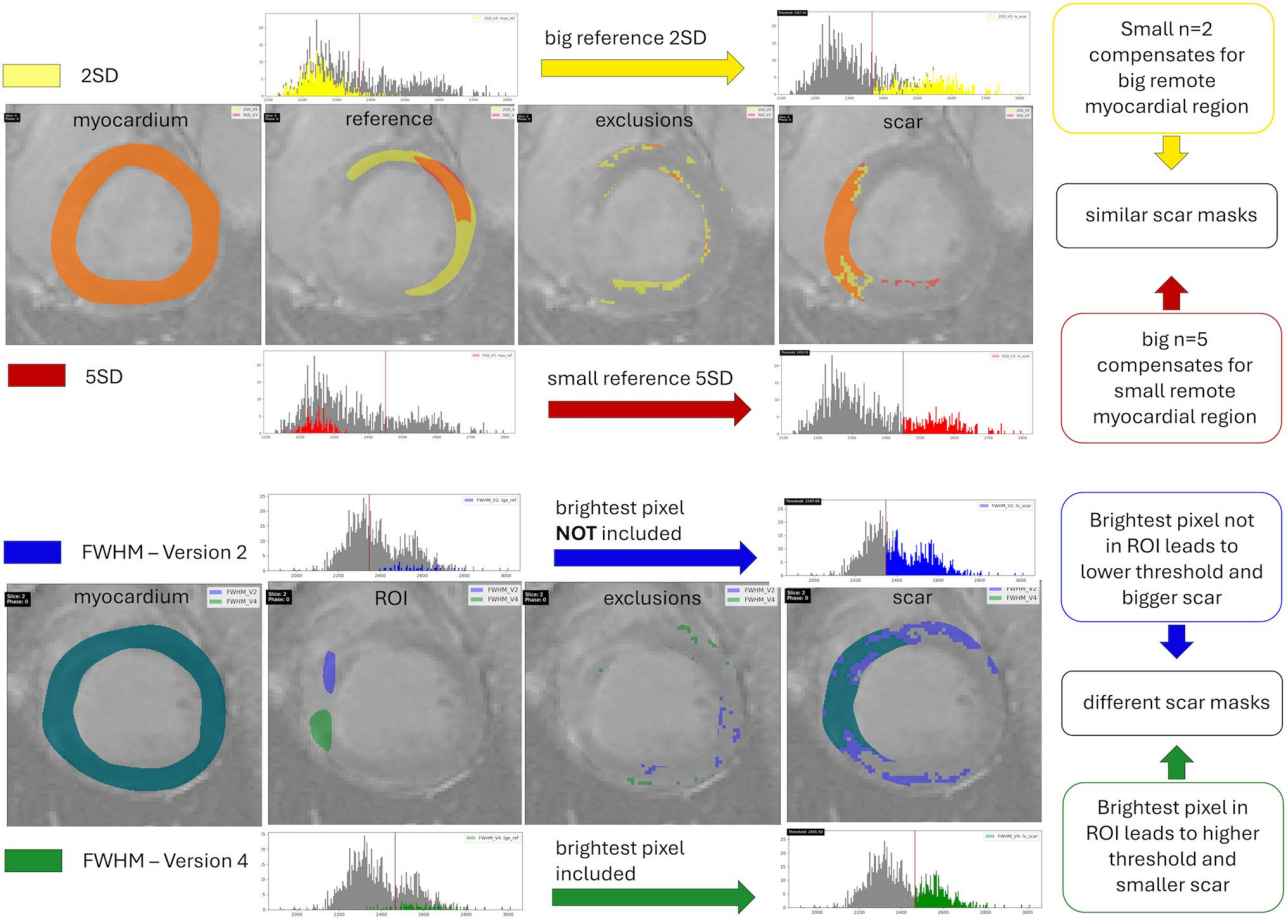
How different regions of interest for the FWHM and different remote myocardial regions for the *n*SD method on the same myocardial annotation can influence the scar masks. The upper part of the figure shows how different sizes of the remote reference with different standard deviations can be compensated by using a higher or lower number *n*. For bigger reference and accordingly larger standard deviation, a smaller number (*n* = 2) needs to be used to calculate a similar threshold than when using a smaller reference and smaller standard deviation accordingly with a higher number (*n* = 5) and generate a similar scar mask. The lower part of the figure shows how different ROIs for the FWHM method can lead to different thresholds and different scar masks accordingly

Different remote myocardial regions were used for the method 2SD V5 und 5SD V3 (Fig. 5). Although the 2SD’s remote myocardial region was roughly three times the size of the 5SD’s remote myocardial region, similar thresholds were calculated throughout the case, which add up to similar scar percentages. The higher standard deviation of the bigger 2SD ROI is mitigated by using a lower *n* = 2. The lower SD of the 5SD method on the other hand is compensated by the higher *n* = 5. However, it is worth noting that the 2SD method required more exclusions than the 5SD method (Fig. 5).

#### Influence of the Placement of the ROI

Figure 5 also shows the impact of utilizing different ROIs for the FWHM method. In this exemplary slice, vastly different scar sizes were calculated after utilizing different ROIs, one which included the brightest pixel of the myocardium and one which did not. Utilizing a ROI which did not include the brightest pixel of the myocardium led to a lower threshold and a bigger scar than utilizing a ROI which included the brightest pixel of the myocardium. Adding up these differences throughout the case, this led to almost double the scar percentage for the FWHM method using only ROIs which did not include the brightest pixel of the myocardium (49.8%) compared to the FWHM method using ROIs which included the brightest pixel of the myocardium (26%).

#### Influence of the Myocardial Annotation

**Fig. 6:**
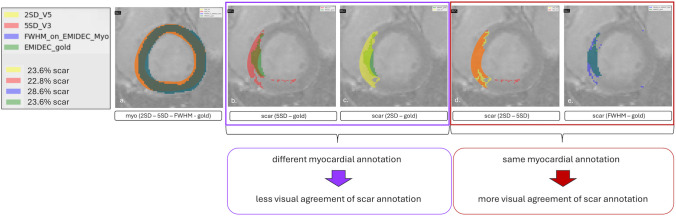
Effects of the myocardial annotation on the deducted scar and the visual agreement between the resulting scars. Depicted are the methods 2SD V5 utilizing a big reference and 5SD V3 utilizing a small reference on the same myocardial annotation, and a semiautomatic FWHM method and the EMIDEC gold scar annotations utilizing the same myocardial annotation. **a** Myocardial annotations of the four methods, **b** scar of 5SD V3 and EMIDEC gold, **c** scar of 2SD V5 and EMIDEC gold, **d** scar of 2SD V5 and 5SD V3, and **e** scar of FWHM semi-automatic approach and EMIDEC gold. The purple box highlights the lower agreement of the scar masks between methods utilizing different myocardial annotations, and the red box highlights the higher agreement of the scar masks between methods utilizing the same myocardial annotation

For this illustration case, the scar percentage for the 2SD V5 method is exactly the same as the scar percentage supplied by the EMIDEC gold annotations (23.6%). When doing a quantitative assessment of the individual slices in the case tabs however, the visual agreement of the scar masks for 2SD V5 and EMIDEC gold is lower than expected (compare purple box in Fig. 6). This is due to the different myocardial annotations used as groundwork. Similar effects can be seen for the 5SD V3 method which also resulted in a similar scar percentage to the EMIDEC gold (22.8% for 5SD vs. 23.6% for EMIDEC gold). A higher visual agreement is given when comparing scar masks based on the same myocardial annotations, as can be seen in the red box of Fig. 6. The scar masks of 2SD V5 and 5SD V3 using the same myocardial annotations have a high agreement, the same is true for the EMIDEC gold and the FWHM semiautomatic method based on the EMIDEC myocardial annotations.

### Application Experiment

Lumos backend calculated the implemented clinical results; the numbers for the different methods per case can be seen in Table using the scar percentage as an example. An extensive table with all calculated clinical results can be seen as supplementary material 2.

The colored cells in Table 4 indicate which thresholding method had the highest agreement with the EMIDEC gold annotations. Note that the scar percentage that most closely agreed with the gold standard is distributed between all methods but 2SD (i.e. FWHM, 3SD-7SD). The highest excluded volume was reached for the 2SD method (compare supplementary material 2).

**Table 4:**
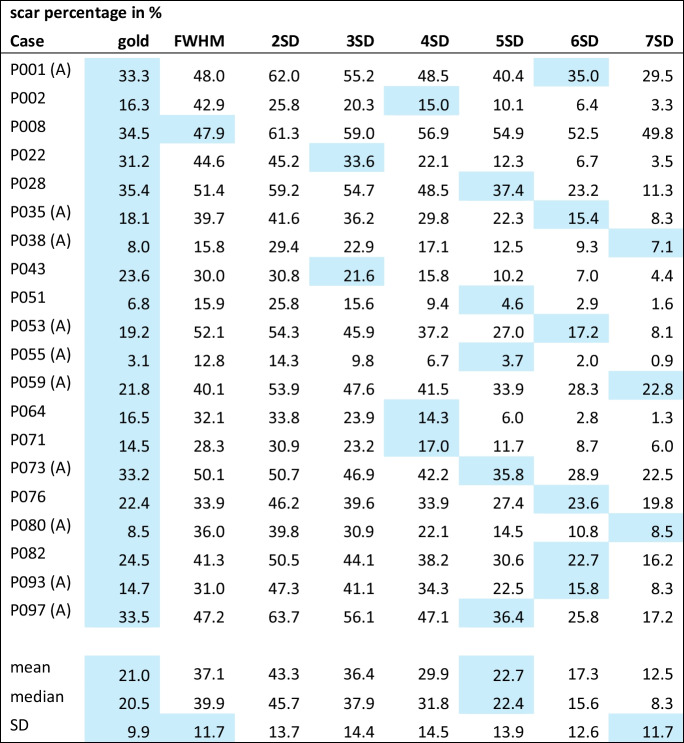
The scar percentage as an exemplary clinical result for the methods gold, FWHM, and 2SD-7SD. Color coded for the closest scar percentage to the gold annotation. The cases labeled with (A) include artifacts

### Statistical Tab: Find Trends And Outlier

The statistical tab shows multiple boxplots below each other for the different methods respectively. By being able to include and exclude cases in the statistics, the cases can be sorted by artifacts (A), or no artifacts (N), and the results can be depicted in the statistical tab respectively. Multiple boxplots for each clinical result can be displayed.

**Fig. 7:**
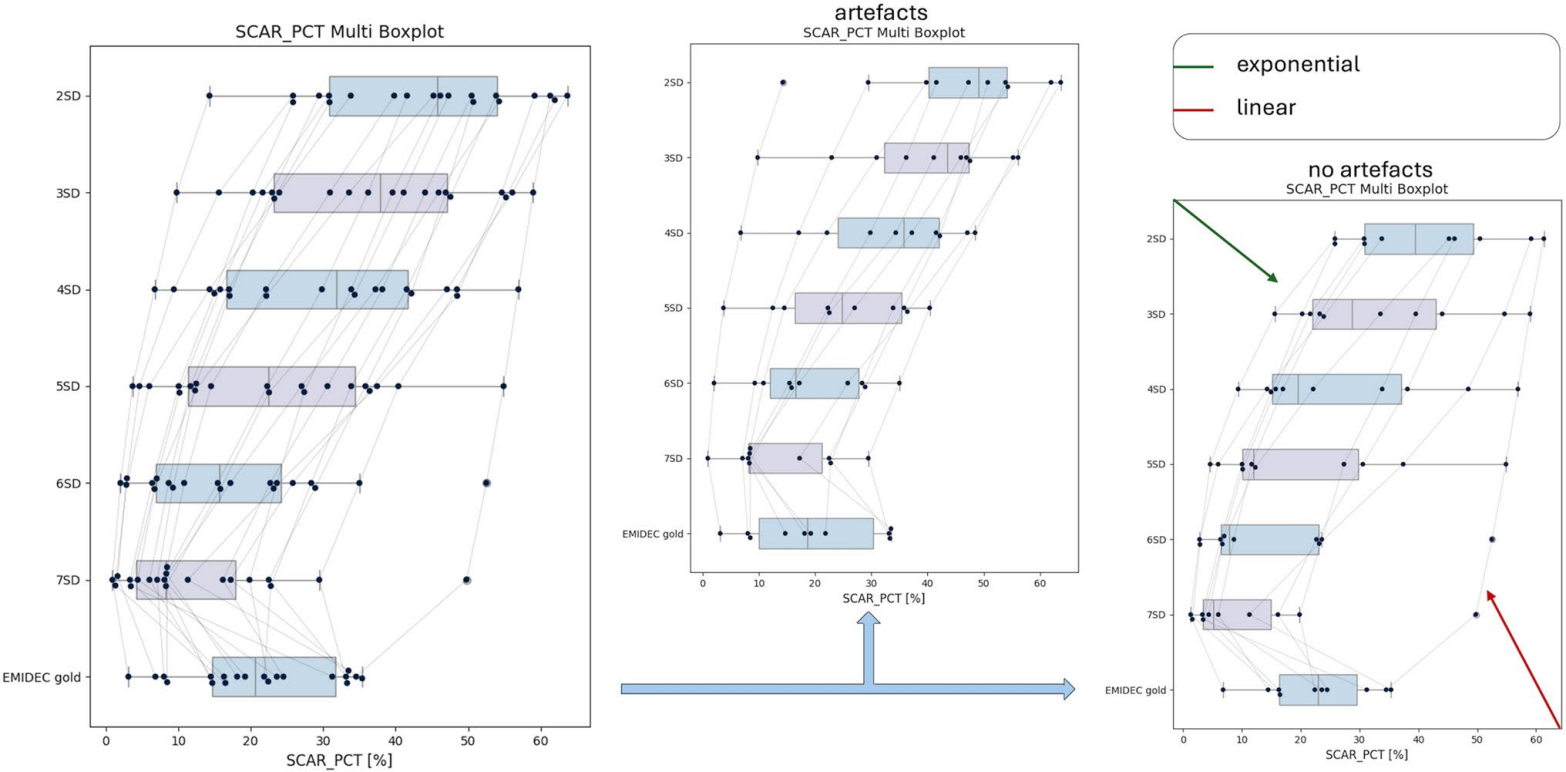
Boxplots for the methods 2SD-7SD and EMIDEC gold offered by the statistical tab. On the left-hand side, the multiple boxplots include all cases; in the middle, only the cases with artifacts are included; and on the right-hand side, cases without artifacts are included. Note that the user can track the cases throughout the methods by the lines connecting the case points; here, for different *n* in the nSD method, the changes in the scar follow a linear decline for the case marked by the red arrow (bottom right) and an exponential decline for the case marked by the green arrow (top left)

In Fig. 7, the multiple boxplots of the scar percentage are shown; on the left-hand side for all cases; in the middle, only cases with artifacts were included; and on the right, only cases without artifacts were included. The displayed methods here are 2SD-7SD and EMIDEC gold.

For the *n*SD methods, it is clearly visible how the scar mass and percentage decrease as *n* increases. The lines between the respective cases show if the changes between the different *n* follow a linear or exponential decline. The shift of the median for the nSD methods is also visible in the boxplots, from bigger median when only artifacts are included to a smaller median when only cases without artifacts are included. In the same way, the different distributions of the cases can be seen by the positions and wideness of the quartiles.

The clinical results in Table 5 support what was seen in the boxplots: The mean and median scar percentage for the *n*SD methods is higher for cases including artifacts than for cases not including artifacts. For the FWHM method and the EMIDEC gold annotations, the differences between cases including artifacts and cases not including artifacts are smaller.

**Table 5:** Mean, median, and standard deviations for the scar percentage. First for all cases and then divided by artifacts (A) and no artifacts (N)

**Scar percentage in %**								
	**gold**	**FWHM**	**2SD**	**3SD**	**4SD**	**5SD**	**6SD**	**7SD**
mean	21.0	37.1	43.3	36.4	29.9	22.7	17.3	12.5
median	20.5	39.9	45.7	37.9	31.8	22.4	15.6	8.3
SD	9.9	11.7	13.7	14.4	14.5	13.9	12.6	11.7
mean (A)	19.3	37.3	45.7	39.3	32.7	24.9	18.9	13.3
median (A)	18.7	39.9	49.0	43.5	35.8	24.8	16.5	8.4
SD (A)	10.6	13.1	14.3	13.9	13.0	11.4	9.8	8.6
mean (N)	22.6	36.8	41.0	33.6	27.1	20.5	15.7	11.7
median (N)	23.0	37.6	39.5	28.8	19.6	12.0	7.9	5.2
SD (N)	8.8	10.2	12.6	14.4	15.5	15.6	14.6	14.0

Additional trends for the *n*SD methods when differentiating between cases including artifacts and not including artifacts can be seen in the statistical results provided by Lumos on the example of the 2SD method (results in format mean ± standard deviation), where the exclusion volume was higher for artifacts (7.8 ± 3.0 ml) than for no artifacts (4.8 ± 3.1 ml) whereas the size of the remote myocardial reference added up through all slices per case was smaller for artifacts (1131.7 ± 267.3 mm^2^) than for no artifacts (1569.7 ± 802.2 mm^2^, compare overview table provided in the supplementary material 2). This shows that if bright artifacts are present, they can be detected as scar tissue by the thresholding method and need to be manually removed afterwards, making more exclusions necessary. At the same time, it is harder to find healthy dark myocardium for the remote reference of the nSD method when artifacts make healthy tissue appear brighter.

### Difference Tracing: Combining Statistical and Case Specific Information

By combining the statistical information with the case-specific information, Lumos can be utilized for tracing differences. By tracking a case through the line between the multi boxplots depicted in Fig. 7, outliers can be found. Clicking on the case point automatically opens the case tabs, where additional information can be gained by regarding the provided case-specific information.

**Fig. 8:**
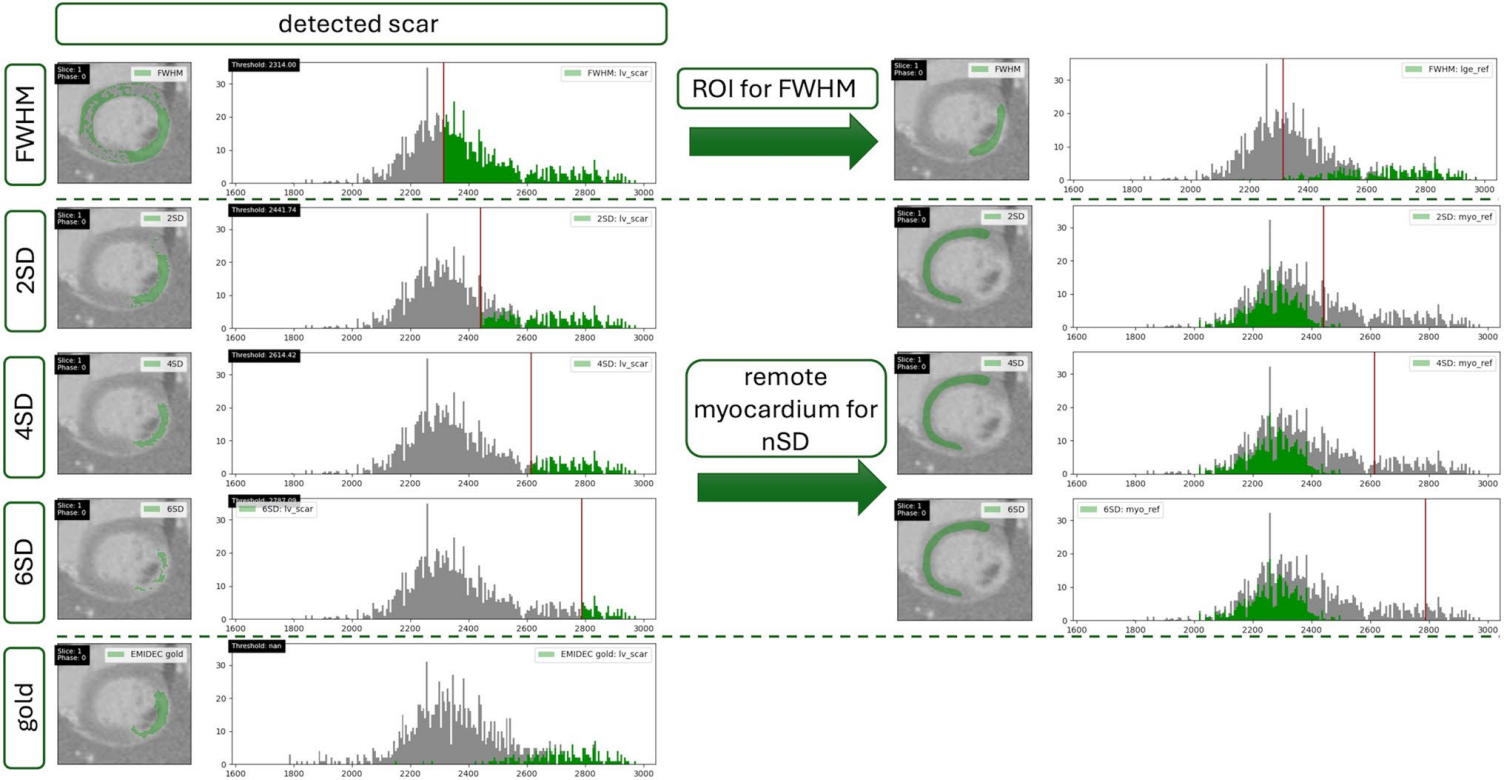
Exemplary slice of a case for the FWHM, 2SD, 4SD, 6SD, and the gold annotation (from top to bottom). One can choose which contour type should be colored in the myocardial histogram. On the left, the detected scar is shown; on the right, the underlying ROI or remote myocardial contour for the thresholding methods. The red vertical line in the histograms represents the threshold

As can be seen in Fig. 8, the histogram case tab provides slice-by-slice figures where on the left-hand side, the selected annotation for each method is plotted onto the image, whereas on the right-hand side, the myocardial histograms of the included pixel intensities are plotted. Highlighted in color is the distribution of the pixel intensities of the selected annotation. The threshold is marked by the vertical red line in the histograms. In Fig. 8 is shown how the user can visually assess the threshold position and confirm if the valley between the distributions was met as intended by the thresholding methods. The user can move through the slices belonging to the selected case. In the exemplary slice in Fig. 8, the valley was met for 4SD, and the distribution looks similar to the distribution provided by the EMIDEC gold standard, whereas for 2SD and FWHM, the thresholds appear to be too far left and for 6SD too far right. When selecting the ROI or remote myocardial reference annotations that were used as a basis for the thresholds, note that for the FWHM method, the ROI included the brightest pixel in the myocardium, but since the darkest pixel partially included in the myocardial histogram has an intensity close to 1650, the calculated threshold is smaller than anticipated. The remote myocardial reference annotation is distributed in the darker half of the myocardial histogram with mean and standard deviation close with those of the distribution representing the healthy myocardium. This explains why 2SD overestimates, 6SD underestimates, and 4SD fits the gold standard most closely.

**Fig. 9:**
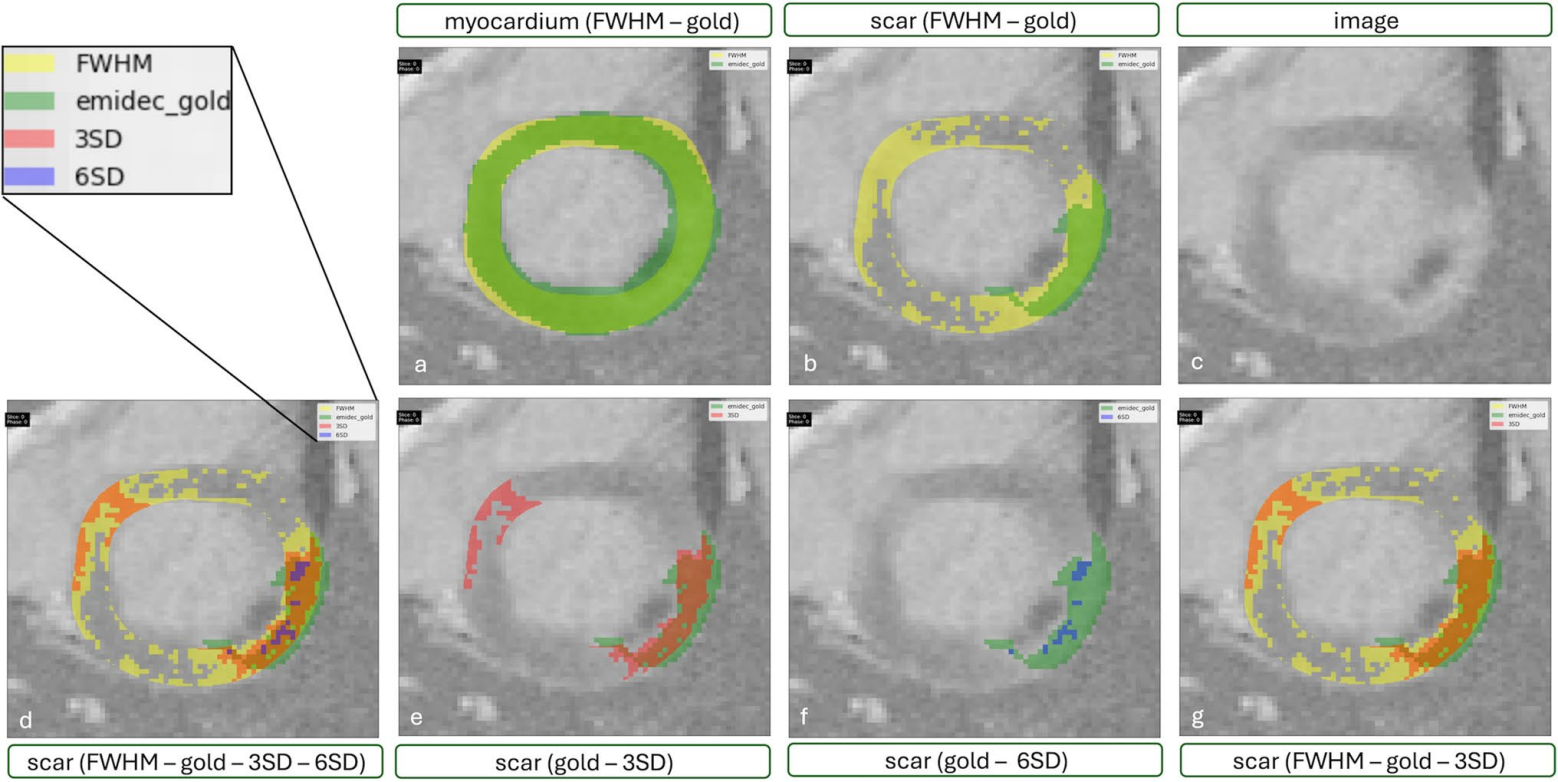
Different annotations plotted onto the dicom image: **a** myocardial annotations of FWHM (yellow) and EMIDEC gold (green); **b** scar annotations of FWHM (yellow) and EMIDEC gold (green); **c** image without additional annotations; **d** scar annotations of FWHM (yellow), EMIDEC gold (green), 3SD (red), and 6SD (blue); **e** scar annotations of EMIDEC gold (green) and 3SD (red); **f** scar annotations of EMIDEC gold (green) and 6SD (blue); **g** scar annotations of FWHM (yellow), EMIDEC gold (green), and 3SD (red)

For a visible assessment of how the annotations compare to each other, the user can look at the case tabs where annotations of multiple methods are plotted onto the same image. The user selects which annotation type should be plotted onto the image. For each case, the user can move through the respective slices and compare the annotations visually. By being able to switch methods on and off, the user can compare as many methods at the same time as needed. In Fig. 9, exemplary annotations are shown: In Fig. 9a, the myocardial annotations for the gold and FWHM method were plotted and in Fig. 9b the scar for the same methods. In Fig. 9d, the scar annotations for FWHM, gold, 3SD, and 6SD are plotted; in Fig. 9e gold and 3SD; in Fig. 9f gold and 6SD; and in Fig. 9g gold, FWHM, and 3SD. Note that the colors of the annotations for each method stay consistent while switching them on and off.

For the metrical comparison, it is possible to go back to the two-reader comparison tabs by choosing the tab and two methods. There, additional information is offered by the Dice coefficient and the Hausdorff metric per slice, area, and milliliter differences and threshold differences.

## Discussion

Lumos successfully provided a multi-level multi-reader comparison for LGE quantification while simultaneously providing insight into how different thresholding methods lead to different scar quantifications. By utilizing the statistical tab and the procured clinical results, it was shown how outliers and trends between methods can be found leading to a better understanding of the methods. Since the case tabs supply additional information such as the position of the thresholds throughout a case or the distribution of the underlying ROI or remote myocardial reference respectively, one can track the differences in the clinical results (e.g., the scar size) back to the causative confounders. In addition, the case tabs offer the possibility to visually assess the agreement between the methods. The application experiment and illustration case showcased the different features Lumos offers which can benefit in the analysis of clinical studies. Since the application was intended to demonstrate Lumos usability, varying beginners’ annotations were helpful to demonstrate how Lumos can reveal pitfalls and misjudgments. This use case was not intended as a clinical study, the results are not clinically relevant, and the number of cases is statistically insignificant. However, it shows how Lumos could aid in upcoming clinical studies.

The division between cases with and without artifacts showed another benefit of Lumos. Though a small number of cases were used, it became apparent that more exclusions were necessary for cases including artifacts and that the size of the remote myocardial region for the *n*SD method is smaller when artifacts are present. That was expected since artifacts are normally brighter than normal myocardium; thus, the area to put the myocardial reference in shrinks and the bright pixels will be assigned to the scar by the algorithm and must be manually excluded afterwards.

Since there is currently no gold standard but a variety of different approaches, it was important to offer the multi-reader comparison. This way, it is possible to compare multiple methods at the same time instead of only one method to the gold standard. The multi reader comparison also aids in finding trends and tracing outliers back to the sources the differences originate from. The inclusion of the histograms as additional visualization was an important part of the difference tracing and to better understand the confounders of the different thresholding methods. Since the thresholding methods FWHM and nSD utilize the distribution of the pixel intensities as a basis for the threshold calculation and thus the generation of the scar, by including the histograms into Lumos, the methods and their confounders can be understood better. The user is able to see the different thresholds in the myocardial histogram and is thus able to directly evaluate if the threshold was chosen as expected. For unexpected thresholds, Lumos can be utilized to backtrack to factors which influenced the threshold calculation.

In addition to being able to compare different methods, it is also possible to compare the same method for different readers or different ROIs or remote myocardial regions as well. By defining tasks, inter- and intra-reader comparisons were included and can also be looked at simultaneously.

Since the metrical comparison is complicated to apply to more than two methods at the same time, Lumos offers visual comparison on the multi comparison level only.

The visualization of different ROIs or remote myocardial references offered by Lumos and seen in the illustration of annotation variations could be used to track the robustness of methods regarding different groundwork (different myocardial annotations or different placed/sized ROIs/references) or regarding different readers. When training CMR beginners, the knowledge gained by using Lumos could be helpful to avoid annotation mistakes.

Lumos code can be found on GitHub in the open repository https://github.com/thequadsquad/Lumos. The code can be adjusted to the user’s needs. By making Lumos openly available as a research tool, the objective is to contribute to the reproducibility and stability of scar quantification methods. With this, as well as sensitizing CMR beginners to potential pitfalls and understanding confounder impacts, Lumos has immense potential to aid in the clinic as a research tool.

The illustration of varying annotations and their effect on clinical results also emphasized the importance of multiple levels when comparing results. By being able to not only compare the scar percentage statistically but also consider individual slices, the evaluation of the trustworthiness of clinical results can be achieved. The exact rasterization approach was necessary for exact calculations with the annotations since the effects of differing annotations could impact the clinical parameters as seen in the illustration case. Especially for the FWHM method, it was important to accurately find the brightest pixel intensity, which is part of the ROI and the darkest pixel intensity, which is part of the myocardium respectively. If the rasterization algorithm did not transfer the polygons into masks exactly, big mistakes in the generation of the threshold and consequently of the scar would be acquired. By implementing an exact rasterization algorithm, no information is lost, neither which pixel intensities are found inside the polygon nor the weight with which the pixel intensity contributes to the respective structure.

Correct segmentation is the basis of most quantification tasks in CMR [1]. The task of annotating cardiac structures is a tedious and laborious task for clinicians and researchers alike [34]. In addition, inter and intra reader variabilities are not only problematic in LGE scar quantification [35]. Artificial intelligence (AI) could automate this process, giving clinicians more time for diagnostics. In LGE scar quantification especially, algorithms not basing on a ROI or a remote myocardial region might also eliminate confounders [14, 36]. There are many machine learning or deep learning algorithm available for segmentation tasks, and a lot already perform fairly well with the DSC [37]. Challenges of automatic segmentation that are left are the segmentations in the base and the apex [37].

As AI plays an increasingly important part in CMR, one objective was to offer the possibility to compare AI generated scars with the mainly used methods at the same time. Lumos as a multi-reader comparison tool will be especially useful as one can not only compare the AI generated scars to one method but to several at the same time. Since there is no established gold standard for scar quantification that could ensure to not build an AI which makes similar mistakes as the commonly used methods, and it could aid to not dismiss an AI too early because they do not agree with only one of the methods. The exact rasterization algorithm should be helpful as well when comparing AI generated annotations since they might not necessarily fit the same subpixel resolution as manually generated annotations. Another use case for Lumos in AI development could be to compare versions of the AI during the development progress with the versions from intermediate steps. This way, developers would be able to track changes of the AI results through the development process. Similar color-coded tables like Table 1 could be helpful for this.

In addition to that, AI could also be utilized to improve the image quality of LGE images [13]. Since different methods might vary in how well they perform on these images, Lumos could be helpful in comparing multiple methods at once and evaluating their performance.

Lumos was built for easy extendibility. As a proof of concept for the extendibility the cine short-axis view and cine long-axis two- and four-chamber views were implemented into Lumos. The views obtained the same statistical tab with multi boxplots and the case tab where multiple annotations can be compared at once and the same go back buttons for a metrical two-reader comparison. In addition to moving through the slices of a case, for the cine view, it is also possible to move through the different phases. This was tested for three cases which included cine images for the short-axis view as well as the two-chamber and four-chamber long-axis view with three methods applied (including two AI generated segmentations).

The software was developed by using phase-sensitive inversion recovery (PSIR) images but should also be tested for magnitude inversion recovery (MagIR) images as well as for more AI generated annotations and annotations generated by different software vendors.

### Limitations

The data used to demonstrate Lumos features has a small number of cases (*N* = 20). The number of readers to be compared at once is limited to 20. The number could be increased by adjusting the backend. However, discerning relevant differences is more difficult for an increasing number of overlaid annotations. Image formats like nifty can only be used after being converted to Dicom format which requires programming skills.

### Outlook

For a wider range of functions, the software should be expanded for additional views such as T1 or T2 mapping. Additional views known from Lazy Luna [18, 19] should be implemented to offer the multi-reader comparison in addition to the two-reader comparison. That way, it would be possible to combine Lazy Luna [18, 19] and Lumos into one tool for simplified use.

Lumos also demonstrated the value in being able to differentiate between cases including artifacts and not including artifacts since artifacts have a big influence on the resulting scar sizes and the image quality. For our data, the cases were sorted by hand; however, for studies with higher N, it would be helpful to automatically label artifacts and to be able to sort the data.

## Conclusions

Lumos as a multi-level multi-reader comparison tool for LGE scar quantification offers a wide range of possible applications. The difference tracing is the core feature of the software. Being able to connect the statistical information on trends and outliers with the case specific information aids in evaluating the method for all cases or an individual case. The different visualizations offered by Lumos are essential to better understand the underlying confounders, in particular for the thresholding methods.

## Supplementary Information

Below is the link to the electronic supplementary material.

Supplementary file1 (MOV 104796 KB)

Supplementary file2 (CSV 17 KB)

Supplementary file3 (PDF 2090 KB)
