# Supplementary material for: Lumos: Software for Multi-level Multi-reader Comparison of Cardiovascular Magnetic Resonance Late Gadolinium Enhancement Scar Quantification
Source: J Imaging Inform Med. 2025 Mar 17;38(6):4167–83. doi: 10.1007/s10278-025-01437-2 (PMC12701143; doi:10.1007/s10278-025-01437-2)
Supplement: Supplementary file 3 — Supplementary file3 (PDF 2090 KB) [file 10278_2025_1437_MOESM3_ESM.pdf]

Supplementary Material 3

The following figures give more details on how the myocardium and ROI/myocardial reference were annotated for the five versions V1-V5 of the FWHM and nSD methods in the illustration case respectively.

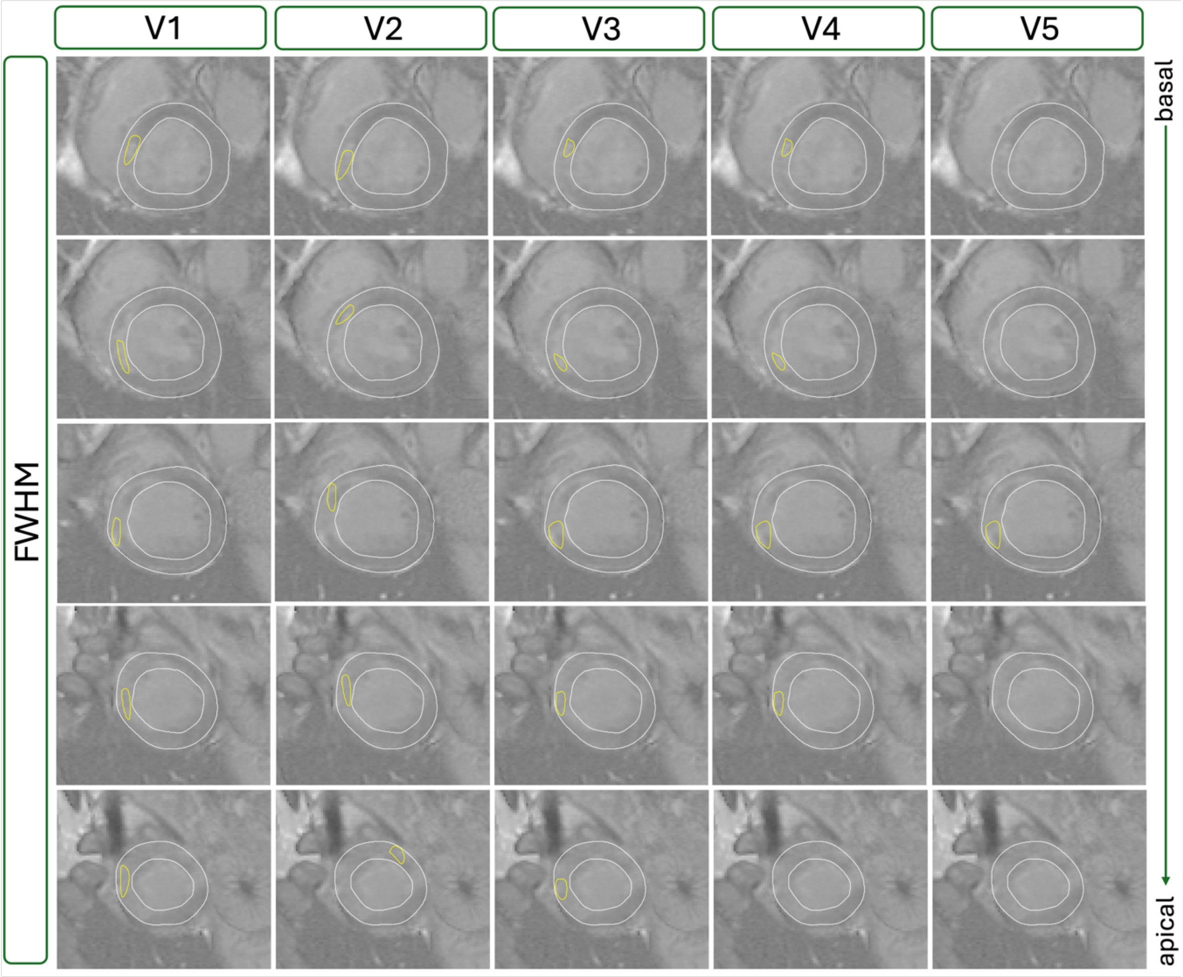

Fig. S1 *For the FWHM method the different locations for the ROI (yellow) in the myocardium (white) are depicted through all five slices from basal (top) to apical (bottom). Version V1-V3 show different positions of the ROI, with ROI placement in all 5 slice, whereas version V4 does not utilize a ROI in the apical slice and version V5 only utilizes one ROI which was annotated in the midventricular slice.*

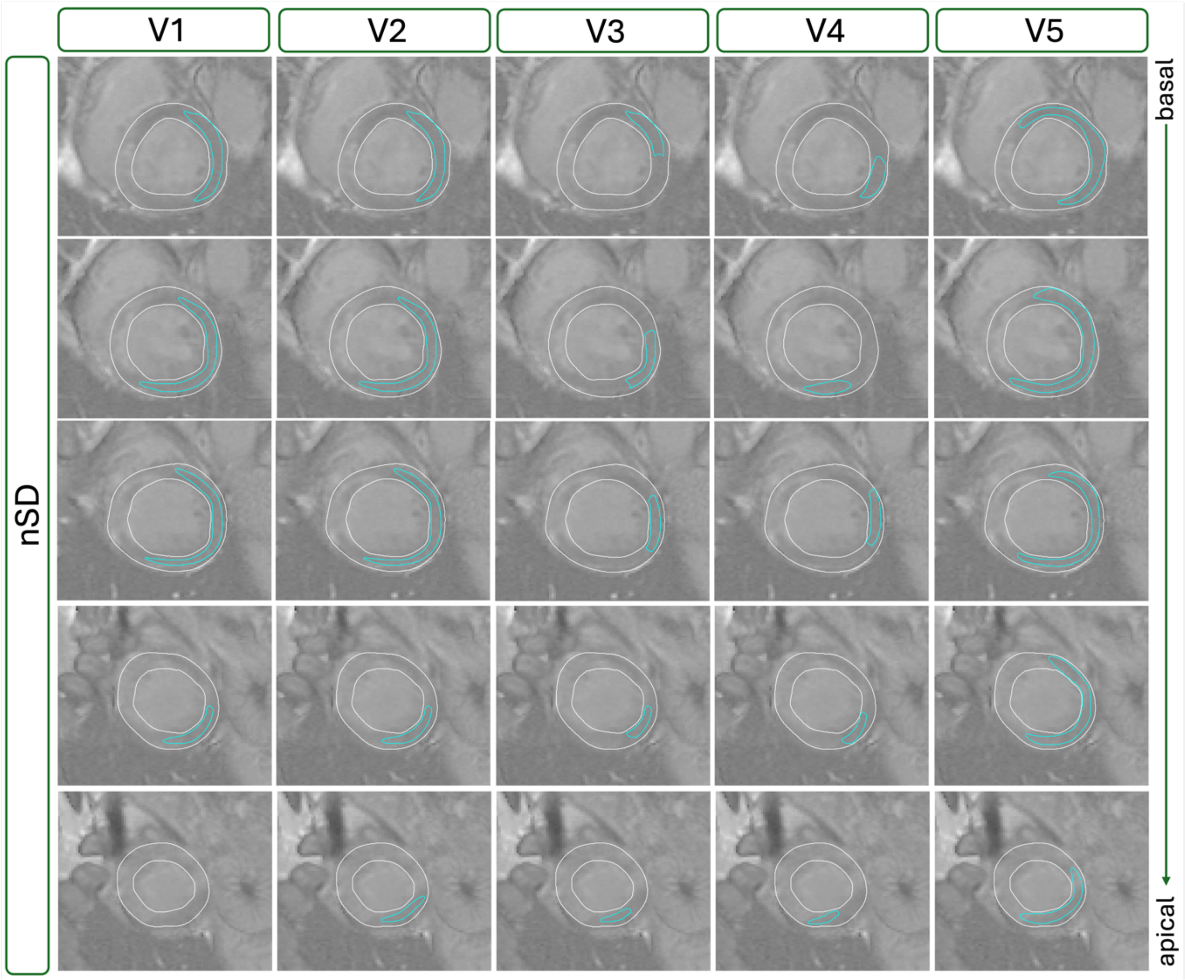

Fig. S2 *For the nSD method the different locations and sizes of the remote myocardial reference (blue) in the myocardium (white) are depicted through all five slices from basal (top) to apical (bottom). Version V1 shows myocardial references in all but the apical slice with sizes approximately ⅓ - ½ of myocardium. Version V2 utilizes the same references with one added one in the apical slice. Version V3 and V4 utilize smaller references (approximately ⅙ - ¼ of myocardium) with different positions.In Version V5 the reference size was increased to at least ½ of myocardium.*
